# Metabolic profiles of biological aging in American Indians: The strong heart family study

**DOI:** 10.18632/aging.100644

**Published:** 2014-03-20

**Authors:** Jinying Zhao, Yun Zhu, Karan Uppal, ViLinh T. Tran, Tianwei Yu, Jue Lin, Tet Matsuguchi, Elizabeth Blackburn, Dean Jones, Elisa T. Lee, Barbara V. Howard

**Affiliations:** ^1^Department of Epidemiology, Tulane University School of Public Health, New Orleans, LA 70112; USA; ^2^Division of Pulmonary, Emory University School of Medicine, Atlanta, GA 30322; USA; ^3^Department of Biostatistics and Bioinformatics, Emory University School of Public Health, Atlanta, GA 30322; USA; ^4^Department of Biochemistry and Biophysics, University of California, San Francisco, CA 94143, USA; ^5^Center for American Indian Health Research, University of Oklahoma Health Sciences Center, Oklahoma City, OK 73104; USA; ^6^Medstar Research Institute and Georgetown and Howard Universities Centers for Translational Sciences, Washington, DC 20007

**Keywords:** metabolic profiles, metabolomics, telomeric age, telomere length, American Indians

## Abstract

Short telomere length, a marker of biological aging, has been associated with age-related metabolic disorders. Telomere attrition induces profound metabolic dysfunction in animal models, but no study has examined the metabolome of telomeric aging in human. Here we studied 423 apparently healthy American Indians participating in the Strong Family Heart Study. Leukocyte telomere length (LTL) was measured by qPCR. Metabolites in fasting plasma were detected by untargeted LC/MS. Associations of LTL with each metabolite and their combined effects were examined using generalized estimating equation adjusting for chronological age and other aging-related factors. Multiple testing was corrected using the q-value method (q<0.05). Of the 1,364 distinct m/z features detected, nineteen metabolites in the classes of glycerophosphoethanolamines, glycerophosphocholines, glycerolipids, bile acids, isoprenoids, fatty amides, or L-carnitine ester were significantly associated with LTL, independent of chronological age and other aging-related factors. Participants with longer (top tertile) and shorter (bottom tertile) LTL were clearly separated into distinct groups using a multi-marker score comprising of all these metabolites, suggesting that these newly detected metabolites could be novel metabolic markers of biological aging. This is the first study to interrogate the human metabolome of telomeric aging. Our results provide initial evidence for a metabolic control of LTL and may reveal previously undescribed new roles of various lipids in the aging process.

## INTRODUCTION

Aging and age-related metabolic disorders, such as obesity, diabetes, and cardiovascular disease (CVD), pose great social and economic burdens worldwide. The aging process is characterized by progressive metabolic decline over time, such as metabolic rate decline [1], reduced insulin secretion and β-cell dysfunction [2, 3], as well as glutathione metabolism [4]. Biological pathways known to be involved in aging are also implicated in metabolism. Therefore, profound metabolic abnormality represents a hallmark of aging [5]. With the current increase in life expectancy and the heavy burden of age-related disorders, there is an urgent need to elucidate metabolic pathways associated with aging and to develop novel metabolic markers and therapeutic targets for early aging and age-related disorders.

Telomeres are repetitive DNA sequences and associated proteins at the end of each chromosome. They are critical in maintaining chromosomal stability and normal aging [6]. Telomere length shortens progressively during each round of cell cycle and declines significantly with age, thus has been emerging as a valuable marker for biological aging and age-related disorders. Progressive metabolic deterioration and telomere-induced biological aging have been shown to be two intimately linked biological processes [7]. On one hand, telomere dysfunction impairs metabolic function [8], and telomerase reactivation reverses tissue degeneration in aged telomerase-deficient mice [9]. On the other hand, metabolic signatures associated with natural aging accurately predicts biological aging provoked by accelerated telomere shortening, and a derived metabolomic score reliably predicts the age of wild-type mice [10]. Moreover, treatment with telomerase reverse transcriptase (TERT) reverses some of the metabolic changes associated with aging [11], further substantiating the association between telomeric aging and metabolic dysfunction. Shortened telomere length has been associated with a variety of metabolic disorders, such as obesity [12], diabetes [13], insulin resistance [14], impaired glucose tolerance [15], atherosclerosis [13, 16], dyslipidemia [17], and hypertension [18]. In a recent study, we reported that leukocyte telomere length at baseline significantly and independently predicts incident diabetes in American Indians [19], lending further support for a strong relationship between biological aging driven by telomere shortening and metabolic dysregulation.

Metabolomics is an emerging high-throughput analytical technology that can measure numerous endogenous and exogenous metabolites in biofluids (e.g., plasma, serum, urine). Because small metabolites are intermediates and end products of all regulatory pathways, metabolic alterations represent the most proximal reporters of alterations in our body in response to intrinsic and extrinsic perturbations, and thus may capture the complex physiological or pathological changes that accompany the aging process [20]. In previous metabolomics studies, changes in serum or plasma metabolites have been associated with chronological age or aging in animal and human studies. For example, several groups reported decreased serum carnitines, acylcarnitines and amino acids with chronological age and increased free fatty acid levels in aging rodents [21]. In human studies, serum carnitine [22], glycerophosphocholines and sphingomyelins increased with chronological age [23]. These investigations demonstrated that metabolic profiles are age-dependent, and metabolomic approaches can be used to capture the metabolic signatures of aging process. However, existing studies focused on metabolic changes related to chronological age, which could be very different from that of biological aging driven by telomere shortening. The goal of this study is to identify metabolic profiles of telomeric aging independent of chronological age and other aging-related factors in American Indians, a minority group suffering from disproportionately higher rates of age-related metabolic disorders, especially type 2 diabetes.

## RESULTS

Table 1 presents the characteristics of study participants according to LTL tertiles. Compared to participants with longer LTL, those with shorter LTL were significantly older, and had significantly higher levels of BMI, waist circumference, LDL-c and total cholesterol as well as eGFR. No significant difference was observed for other listed variables across LTL tertiles.

**Table 1:** Characteristics of the SHFS study participants according to LTL tertiles (n=423)

T/S ratio	Tertile 1 (n=139 )	Tertile 2 (n=144 )	Tertile 3 (n=140 )	P trend*
Mean	0.7549±0.1281	0.9960±0.0511	1.2584±0.1783	
Median	0.7865	0.9988	1.2121	
Interquartile range	0.6684-0.8565	0.9521-1.0411	1.1339-1.3171	
Age (years)	39.50±14.33	33.87±12.24	28.37±11.16	<0.0001
Female (%)	65.47	68.06	61.43	0.4787
BMI (kg/m^2^)	34.08±7.72	32.56±8.36	32.01±9.08	0.0488
WC (cm)	107.46±17.83	103.58±19.12	101.44±20.08	0.0081
Current smoker (%)	30.93	40.97	35.71	0.1891
Current drinker (%)	68.35	65.97	67.14	0.5926
SBP (mmHg)	119.65±13.67	120.18±13.93	118.46±13.92	0.4777
DBP (mmHg)	76.06±9.40	76.47±11.15	75.75±12.19	0.8109
HDL (mg/dL)	51.19±14.32	50.74±15.36	50.46±14.57	0.6785
LDL (mg/dL)	101.41±30.90	98.12±28.13	93.37±27.48	0.0210
Total triglyceride (mg/dL)	143.67±74.09	149.11±92.95	136.51±68.94	0.4131
Total cholesterol (mg/dL)	180.69±35.55	178.18±33.31	170.77±32.14	0.0146
Fasting glucose (mg/dL)	91.62±7.15	90.69±7.56	90.72±6.93	0.2702
Fasting insulin (uU/mL)	15.82±9.95	15.93±12.43	16.89±14.47	0.4734
HOMA-IR	3.64±2.46	3.63±2.93	3.78±3.18	0.6999
eGFR	101.44±25.17	103.72±22.94	110.39±22.31	0.0037
Dietary protein (g/d)	93.01±80.35	93.54±78.54	98.44±85.36	0.5130
Dietary fat (g/d)	123.71±97.54	120.09±92.38	127.28±103.00	0.7297
Caloric intake (Kcal/d)	2778.99±2046.01	2729.79±2052.15	2942.66±2154.84	0.4704

* P values were estimated by GEE to account for family relatedness

**Figure 1:**
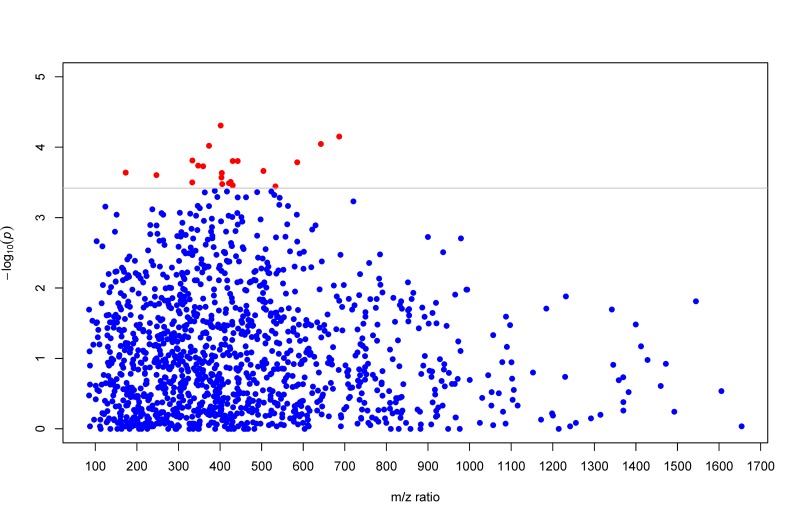
Manhattan plot (−log_10_ *p* vs metabolic feature) showing metabolites using raw *p* values obtained from multivariate GEE regression. Metabolites significantly associated with LTL are highlighted in red dots at the q-value level of 0.05.

A total of 1,364 distinct m/z features matching known metabolites in the current metabolomics databases was included in the present analysis. Of these, altered levels of 19 matching metabolites were significantly associated with LTL after accounting for potential confounders (including chronological age) and multiple testing (at the q-value <0.05 level). Specially, higher levels of 13 metabolites in the species of glycerol-phosphoethanolamines (PEs), glycerophospho-choline (PC), bile acids, fatty amides, L-carnitine ester, peptide, and toluene were significantly associated with longer LTL, whereas higher levels of glycerolipids, glycerophosphoglycerol, isoprenoids, and steroids were significantly associated with shorter LTL. We also estimated the joint effects of risk or protective metabolites on LTL variation using multi-marker metabolites scores comprising of all risk or protective metabolites, respectively. On average, per 10% increase in the multi-marker score comprising of all six risk metabolites was associated with 0.94% shorter in LTL (T/S ratio). By contrast, per 10% increase in the multi-marker score of all thirteen protective metabolites was associated with 0.79% longer in LTL (T/S ratio). Multivariate associations of each individual metabolite and their combined effects with LTL are shown in Table 2. For ease of visual inspection, Figure 1 shows a Manhattan plot (−log_10_ *p* vs metabolic feature) of all metabolites using raw *p* values obtained from multivariate regression analysis. Metabolites significantly associated with LTL are shown at the level of q-value 0.05.

**Table 2:** Association of the detected metabolites (q-value <0.05) with leukocyte telomere length by multivariate GEE*

Matching metabolites	Class	m/z	Retention time	Effect size (95% CI)^†^	P-value
*Protective matching metabolites*					
PE(O-18:0/13:0)	Glycerophosphoethanolamine	686.501	439.45	0.48 ( 0.25, 0.71)	9.26×10^−5^
PE(P-16:0/12:0)	Glycerophosphoethanolamine	642.452	435.46	0.51 ( 0.25, 0.77)	4.92×10^−4^
PC(O-8:0/O-8:0)	Glycerophosphocholine	504.340	19.88	0.63 ( 0.39, 0.88)	2.17×10^−4^
Norchenodeoxycholic acid	Bile acids and derivatives	401.263	297.93	0.41 ( 0.18, 0.63)	2.71×10^−4^
5β-Chol-2-en-24-oic Acid	Bile acids and derivatives	359.295	404.96	0.83 ( 0.49, 1.16)	1.87×10^−4^
3α,6β,7α,12β-Tetrahydroxy-5β-cholan-24-oic Acid	Bile acids and derivatives	425.288	28.14	0.58 ( 0.30, 0.85)	3.10×10^−4^
5β-Cholestane-3α,7α,24-triol	Bile acids and derivatives	421.365	508.16	0.65 ( 0.35, 0.94)	3.23×10^−4^
p-Cresol sulfate (PCS)	Toluenes	173.029	20.11	0.56 ( 0.30, 0.82)	1.45×10^−4^
Dimethylallyl pyrophosphate	Isoprenoid	247.011	42.92	0.61 ( 0.31, 0.92)	2.48×10^−4^
Leu-Ala-Val-Ala (LAVA)	Tetrapeptide	373.243	320.09	0.28 ( 0.08, 0.48)	3.19×10^−4^
N-arachidonoyl histidine	Fatty amides	442.309	345.69	0.68 ( 0.37, 0.98)	1.57×10^−4^
N-palmitoyl phenylalanine	Fatty amides	404.318	518.86	0.72 ( 0.40, 1.05)	2.32×10^−4^
Hexadecanedioic acid mono-L-carnitine ester	Fatty Acid Esters	430.314	575.80	0.64 ( 0.34, 0.95)	3.47×10^−4^
***Combined protective effect***				0.79 ( 0.45, 1.13)	6.54x10^−5^
*Risk matching metabolites*					
MG(20:3)	Glycerolipid	403.279	472.83	−0.49 (−0.79,−0.19)	2.87×10^−4^
DG(18:2/14:1)	Glycerolipid	585.443	556.01	−0.33 (−0.54,−0.11)	1.63×10^−4^
PG(20:4)	Glycerophosphoglycerol	533.289	401.75	−0.62 (−0.90,−0.35)	3.58×10^−4^
(11Z)-8,18-ethanoretinal	Isoprenoid	333.221	275.34	−0.71 (−1.03,−0.39)	1.54×10^−4^
5,6-epoxy-3-hydroxy-5,6-dihydro-12'-apo-β-caroten-12'-al	Isoprenoid	405.243	21.45	−0.80(−1.15, −0.45)	3.32×10^−4^
Corticosterone	Steroid	347.220	77.58	−0.54 (−0.76,−0.31)	1.83×10^−4^
***Combined risk effect***				−0.94 (−1.33,−0.54)	9.57x10^−5^

* Adjusted for age, sex, study center, waist circumference, LDL-c, total cholesterol and eGFR;

† Percent change in LTL (T/S ratio) per 10% change in relative abundance of metabolites

**Figure 2:**
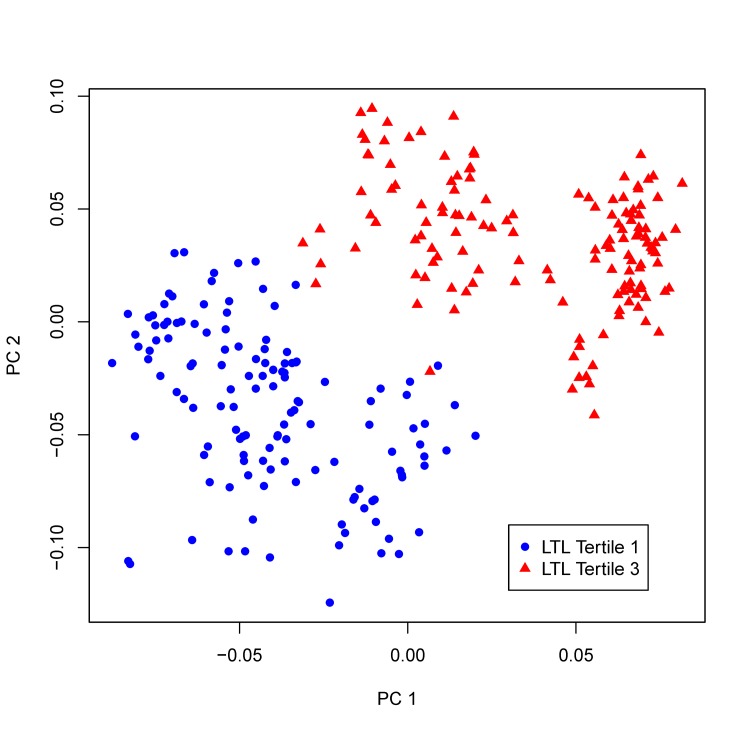
sPLS-DA plot. Participants whose LTL in the top tertile of LTL distribution and those in the bottom tertile are classified into two distinct groups using the multi-marker score comprising of all 19 metabolites significantly associated with LTL in the multivariate GEE model.

Using a multi-marker score comprising of all 19 significant metabolites, we performed sPLS-DA to examine whether these newly identified compounds can discriminate participants with different profiles of biological aging. Figure 2 clearly shows that participants with longer (top tertile) and shorter (bottom tertile) LTL were classified into two distinct groups, suggesting that these newly detected metabolites can be used as biomarkers for risk stratification. Sensitivity analyses showed that additional adjustments for lifestyle (smoking, alcohol drinking), and dietary intake of fat, protein as well as caloric intake did not change our results. Additional adjustment for batch effect in telomere assay also did not attenuate the observed associations.

## DISCUSSION

Using an untargeted high resolution metabolomic approach, here we report significant association of altered metabolic profiles in fasting plasma with telomeric aging among apparently healthy American Indians in the Strong Heart Study. The observed associations were independent of chronological age and many other potential risk factors of aging, and withstood additionally adjustments for lifestyle and dietary factors, suggesting that these newly detected chemicals could be novel indicators of biological aging. As far as we are aware, this is the first study to interrogate the comprehensive metabolome of telomeric aging not only in American Indians but other ethnic groups as well. Findings of this study provide novel insights into telomere biology, and may also facilitate efforts to uncover potential therapeutic targets for anti-aging and aging-related metabolic disorders.

Glycerophosphatidylethanolamines (PEs) are ether-linked phospholipids required for normal developmental, physiological, and cognitive functions [37]. They are proposed to act as antioxidants and may also influence intracellular signaling and membrane dynamics. In the present study, we found that plasma levels of two metabolites matching PE (O-18:0/13:0) and PE (P-16:0/12:0), are positively associated with LTL, suggesting that they may protect against aging. This is in agreement with previous studies demonstrating that PE deficiency was associated with age-related disorders such as Alzheimer's disease [38]. The protective effect of these PEs on aging may be attributable to their antioxidant effects [39]. Glycerophosphocholine (GPC) has been shown to be beneficial on cognitive decline in aging, and choline alphoscerate (a cholinergic precursor) has been widely used in the treatment of neurodegenerative disorders such as Alzheimer's disease [40]. Consistently, we found that a metabolite matching PC (O-8:0/O-8:0), belonging to the class of GPC, was significantly protective on telomeric aging in our study population.

Bile acids, the oxidized derivatives of cholesterol produced in the liver, play an important role in regulating glucose, lipids, and energy metabolism. They are also steroid hormones activating specific nuclear receptors, and have been recognized as signaling molecules of metabolic homeostasis [41]. Previous studies demonstrated that bile acids extended lifespan in model organisms [42, 43], and that bile acid synthesis decreased with aging in human population [44]. Consistent with these observations, we found that higher plasma levels of four metabolites in the class of bile acids and derivatives (norchenodeoxycholic acid, 5β-chol-2-en-24-oic acid, 5β-cholestane-3α,7α,24-triol, and 3α,6β,7α,12β-tetrahydroxy-5β-cholan-24-oic acid) were significantly associated with longer LTL, lending further support for a potential beneficial effect of bile acids on aging. While the precise mechanisms through which bile acids influence aging are unknown, it is possible that they could act as endocrine regulators of aging via nuclear receptor signaling [43] or through influencing oxidative stress and genomic stability [42], all of which may be implicated in telomere maintenance and telomeric aging [45].

In this study, two compounds matching N-palmitoyl phenylalanine and N-arachidonoyl histidine are protective on telomeric aging. These two chemicals belong to fatty amides, which are increasingly recognized as an important new class of lipid signaling molecules. These endogenous signaling molecules have been reported to act as physiological regulators of pain and inflammation [46]. The observed protective effect of these two fatty amides on biological aging in our study could be attributed to their potential anti-inflammatory activities [47].

The p-Cresol sulfate (PCS) is organic solute produced by bacterial metabolism of the amino acids tyrosine in colon. The health effects of free p-Cresol and its conjugates have been inconsistent in previous studies [48, 49]. Here we found that increased level of plasma PCS was associated with longer LTL, suggesting a protective effect on biological aging. Because PCS is metabolized by bacteria in colon, the observed association of PCS with LTL may imply a possible role of gut microbiota in telomeric aging.

Monoacylglycerol (MG) and diacylglycerol (DG) are lipid intermediates believed to be the true lipotoxic culprits underlying the known detrimental effect of triacylglycerol (TG) on insulin resistance [50] and many age-related metabolic disorders, e.g., atherosclerosis and diabetes. In support of this, elevated levels of MG (20:3) and DG (18:2/14:1) were significantly associated with shorter LTL in our analysis. It is possible that these glycerolipids may function as second messengers that impair insulin [51] and/or transmembrane signaling, both of which are known to be involved in the process of aging [52].

The isoprenoid pathway is an important metabolic pathway for the production of dimethylallyl pyrophosphate (DMAPP) and isopentenyl pyrophosphate, which serve as the basis for the biosynthesis of molecules that are essential for a variety of processes such as cell membrane maintenance, hormones metabolism, protein anchoring, and steroid synthesis. The observed associations of telomeric aging with several matching isoprenoids (e.g., DMAPP, (11Z)-8,18-ethanoretinal, and 5,6-epoxy-3-hydroxy-5,6-dihydro-12'-apo-β-caroten-12'-al) in our study are consistent with a *priori* knowledge on the related isoprenoid pathways, though the precise mechanisms underlying these associations await further investigation.

In addition to the altered profiles of lipids, bile acids, and isoprenoids discussed above, altered levels of several metabolites matching corticosterone, mono-L-carnitine ester and tetrapeptide were also associated with LTL. While the molecular mechanisms linking these chemicals to telomere variability remain to be determined, the negative effect of corticosterone on telomere aging observed in our analysis appears to be consistent with previous evidence demonstrating that elevated cortisol levels were associated with cognitive aging in human [53]. In addition, previous studies reported that tissue L-carnitine levels decline with age [54], and feeding aged rats with acetyl-L-carnitine reversed the age-related declines of L-carnitine levels in tissue and also reversed a number of age-related changes in liver mitochondrial function [55]. These results support the beneficial effect of hexadecanedioic acid mono-L-carnitine ester on biological aging observed in our study. The protective effect of tetrapetide (LAVA) on telomere aging in our study appears to be also in line with the anti-inflammatory and antioxidant properties of bioactive peptides observed in previous research [56].

Our study has several limitations. First, although our high-resolution LC-MS detected many distinct features, it should be noted that only 18% of the ions detected had a match in the current metabolomics database. These compounds were unable to be pursed due to the large number of possible isomers and a lack of available standards, however, these currently unannotated metabolites may represent genuine metabolites associated with disease process and with the advancement of metabolomic research, we expect that majority of these unknowns will ultimately be annotated and their associations with disease will be identified. Second, although we were able to control many known risk factors related to aging, the possibility of potential confounding by other factors, such as diet and gut microbiota, cannot be entirely excluded. Third, study participants included in the current analysis are American Indians; it is unclear whether our findings could be generalized to other populations with different genetic and/or lifestyle background. Finally, our results need to be replicated in large-scale metabolomic analysis of American Indians and other ethnic populations as well.

In summary, this is the first study to interrogate the human metabolome of biological aging. Altered plasma levels of nineteen metabolites are significantly associated with interindividual variability in LTL, independent of chronological age and many other aging-related factors. These newly detected metabolites are consistent with known pathophysiological mechanisms of aging and are in agreement with previous studies, suggesting biological plausibility of our findings. Our results provide a better understanding and potential novel markers of telomeric aging. Targeting biological pathways that involve these newly detected metabolites may help to develop preventive and therapeutic strategies towards healthy aging and age-related disorders.

## METHODS

### Study population

The Strong Heart Family Study (SHFS) is a family-based prospective cohort study to identify genetic factors for CVD, diabetes and associated risk factors in American Indians. The study was initiated in 2001-2003 by recruiting 3,665 individuals (14-93 years older) from 94 multi-generational families residing in Arizona (AZ), North and South Dakota (DK) and Oklahoma (OK). Study design and methods of the SHFS have been described previously [24]. The SHFS protocol was approved by the Institutional Review Boards from the Indian Health Service and the participating centers. All subjects gave informed consent.

The current analysis included 423 SHFS participants with complete telomere and metabolomics data. Participants with overt CVD or T2D and those on hypoglycemic (both oral and insulin) medications were excluded from this analysis. The focus on an apparently healthy population will facilitate the identification of early metabolic biomarkers and therapeutic targets for anti-aging and age-related disorders.

### Assessments of risk factors

Fasting plasma glucose, insulin, lipids, lipoproteins and inflammatory biomarkers were measured by standard laboratory methods [24]. Body mass index (BMI) was calculated as weight in kilograms divided by the square of the height in meters (kg/m^2^). Diabetes was defined as fasting plasma glucose ≥7.0 mmol/L and/or treatment for diabetes [25]. Hypertension was defined as blood pressure levels ≥ 140/90 mm Hg or use of antihypertensive medications. Cigarette smoking was classified as current smokers, former smokers and nonsmokers. Alcohol consumption was determined by self-reported history of alcohol intake, the type of alcoholic beverages consumed, frequency of alcohol consumption, and average quantity consumed per day and per week. Participants are classified as current drinkers, former drinkers and never drinkers. Dietary intake was assessed using the block food frequency questionnaire (FFQ) [26]. Level of physical activity was estimated by the mean number of steps per day on pedometer calculated by averaging the total number of steps recorded each day during the 7-day period.

### Measurement of leukocyte telomere length (LTL)

Details for the measurement of LTL have been described elsewhere [19]. Briefly, relative mean telomere length was measured using a high-throughput telomere length assay system designed by Dr. Elizabeth Blackburn's laboratory at the University of California, San Francisco. This assay determines the ratio of telomeric product/single copy gene (T/S) obtained using quantitative PCR (qPCR) according to protocols described previously [27, 28]. The rationale of this method is that the longer the telomeres are in each sample, the more PCR product will be generated in PCR reactions using primers specific for the telomeric DNA. This can be quantified by qPCR using a serially diluted standard DNA and the standard curve method. To normalize the quantity of the input DNA, a single copy gene was amplified in parallel as well. The ratio of the telomeric product versus the single copy gene reflects the average length of the telomeres. Each DNA sample was assayed three times (each time with duplicates), and the three T/S ratios were normalized to the mean of all samples and used in the analysis. For quality control, seven control DNA samples from various cancer cell lines were included in each assay plate. These control samples allowed us to create standard curves, which were then integrated into a composite standard curve used for T and S concentration calculations. In addition, 4.1% of the total sample was assayed in duplicate. Telomere length of the replicate samples were highly correlated (*r* = 0.95, p<0.0001). The intra-assay and inter-assay (assay-to-assay) percentage of coefficient variation were 4.6% and 6.9%, respectively. Lab technicians were blinded to sample duplication and any knowledge of clinical data.

### Metabolic profiling by high resolution liquid chromatography-mass spectrometry (LC-MS)

Relative abundance of fasting plasma metabolites was determined using non-targeted metabolomic approach via high-resolution LC-MS. Detailed lab protocols have been described elsewhere [29, 30]. Briefly, 65 μL plasma sample aliquots were treated with acetonitrile, spiked with internal standard mix, and centrifuged at 13,000 x g for 10 minutes at 4°C to remove proteins. 130 μL supernatant was removed and loaded into autosampler vials. Mass spectral data were collected with a 10 minute gradient on a Thermo LTQ-Velos Orbitrap mass spectrometer (Thermo Fisher, San Diego, CA) to collect data from mass/charge ratio (*m/z*) 85 to 2000 in a positive ionization mode. Three technical replicates were run for each sample using dual column chromatography procedure with C18 and an anion exchange column. Peak extraction, data alignment and feature quantification were performed using the adaptive processing software (apLCMS) [31], a computer package designed for high-resolution metabolomics data analysis. Potential metabolite identities were determined by performing online search (10 ppm mass accuracy) against the Metlin database [32], the Human Metabolomics Database (HMDB)[33], and the LIPID MAPS structure database (LMSD) [34]. Data filtering, normalization, diagnostics and summarization were performed using the computer package MSPrep [35]. Missing data were imputed using the half of the minimum observed value within each metabolite across all samples. Metabolites with high analytical variance (e.g., coefficient variation > 50%) in our samples were excluded from further analyses. Batch-effect was corrected using the algorithm ComBat implemented in MSPrep[35]. Lab technicians were blinded to clinical data of study participants.

### Statistical analysis

Prior to statistical analysis, metabolites data were log-transformed and standardized to unit variance and zero mean (z-scores). Continuous variables such as age, BMI, waist circumference (WC), low-density lipoprotein cholesterol (LDL-c), total cholesterol (TC), eGFR, and fasting glucose were also converted to standard normal distributions with corresponding mean and standard deviation. Tests for linear trends across LTL tertiles were conducted by using the median value in each LTL tertile as a continuous variable in the GEE regression models.

To estimate the effect of each metabolite on telomere variation, we conducted multivariate regression analysis using generalized estimating equation (GEE), which accounts for relatedness among family members. In the GEE model, the level of each matching metabolite (continuous variable) was the independent variable, and telomere length (continuous variable) was the dependent variable, adjusting for chronological age and other clinical factors that differ significantly across LTL tertiles, including WC, LDL-c, TC, and eGFR (see Table 1). Sex and study site were also included in the model. The combined effects of metabolites on telomere length variability were estimated by constructing a multi-marker score based on metabolites that are significantly associated with LTL by fitting a model according to the formula: $\sum_{i}\beta_{i}\;X_{i}$ , where X*_i_* denotes the z-score of the *i-*th metabolite and β_i_ represents the regression coefficient from the GEE regression model containing the indicated metabolites. Given the potential high correlations among many metabolites, we used the q-value method to adjust for multiple comparisons [36], and a q-value < 0.05 was considered statistically significant.

To identify metabolic profiles associated with telomere length, we conducted sparse partial least-squares discriminant analysis (sPLS-DA) using the computer package ‘mixOmics' implemented in the *R* package. The sPLS-DA is a supervised, multivariate technique to determine metabolic groups associated with disease risk. Unlike principal component analysis (PCA) which focuses on variance maximization of the predictors alone, the sPLS-DA models covariance maximization between predictors (metabolites) and disease phenotype (telomere length) when estimating the parameters of a linear regression model, thus represents a regression extension of PCA. The sPLS-DA analysis included only metabolites showing significant associations with LTL and adjusted for age, sex, study center and BMI. For ease of visualization, we presented a Manhattan plot (−log_10_ *p* vs metabolic feature) showing the significance of individual metabolites associated with LTL using raw *p* values obtained from multivariate regression analysis (FDR at *q*=0.05 with a horizontal line). To examine the robustness of our results, we conducted additional analyses by further adjusting for lifestyle factors (smoking, alcohol consumption and physical activity level), diet (dietary fat, protein and caloric intake) and social economic variables.
